# Deep learning for the rapid automatic segmentation of forearm muscle boundaries from ultrasound datasets

**DOI:** 10.3389/fphys.2023.1166061

**Published:** 2023-07-13

**Authors:** Chen Xin, Baoxu Li, Dezheng Wang, Wei Chen, Shouwei Yue, Dong Meng, Xu Qiao, Yang Zhang

**Affiliations:** ^1^ Rehabilitation Center, Qilu Hospital of Shandong University, Jinan, China; ^2^ School of Mathematics, Shandong University, Jinan, China; ^3^ Department of Biomedical Engineering, School of Control Science and Engineering, Shandong University, Jinan, Shandong, China

**Keywords:** ultrasound, image recognition, U-net, deep learning, muscle boundaries

## Abstract

Ultrasound (US) is widely used in the clinical diagnosis and treatment of musculoskeletal diseases. However, the low efficiency and non-uniformity of artificial recognition hinder the application and popularization of US for this purpose. Herein, we developed an automatic muscle boundary segmentation tool for US image recognition and tested its accuracy and clinical applicability. Our dataset was constructed from a total of 465 US images of the flexor digitorum superficialis (FDS) from 19 participants (10 men and 9 women, age 27.4 ± 6.3 years). We used the U-net model for US image segmentation. The U-net output often includes several disconnected regions. Anatomically, the target muscle usually only has one connected region. Based on this principle, we designed an algorithm written in C++ to eliminate redundantly connected regions of outputs. The muscle boundary images generated by the tool were compared with those obtained by professionals and junior physicians to analyze their accuracy and clinical applicability. The dataset was divided into five groups for experimentation, and the average Dice coefficient, recall, and accuracy, as well as the intersection over union (IoU) of the prediction set in each group were all about 90%. Furthermore, we propose a new standard to judge the segmentation results. Under this standard, 99% of the total 150 predicted images by U-net are excellent, which is very close to the segmentation result obtained by professional doctors. In this study, we developed an automatic muscle segmentation tool for US-guided muscle injections. The accuracy of the recognition of the muscle boundary was similar to that of manual labeling by a specialist sonographer, providing a reliable auxiliary tool for clinicians to shorten the US learning cycle, reduce the clinical workload, and improve injection safety.

## 1 Introduction

According to the World Health Organization, about 1.71 billion people worldwide suffer from musculoskeletal disorders (Burton and Kendall, 2014). Musculoskeletal disorders severely limit the mobility and activity of patients, resulting in a reduced quality of life and ability to participate in social activities. Due to population growth and aging, the demand for rehabilitation from musculoskeletal diseases is expected to increase in the coming decades. As such, more instruments are being applied in clinical practice to assist in evaluation and treatment. Ultrasound (US), which uses the principle of reflection and the transmission phenomenon of ultrasonic waves propagated through the human body to obtain images with different echoes (Whittaker and Stokes, 2011), could be applied for various diagnosis and treatment purposes.

US is widely used in various musculoskeletal diseases, such as dystonia, rotator cuff injury, and periarthritis of the shoulder. Using US, clinicians can observe the structure and working relationship of muscles, tendons, ligaments, and other tissues; can identify and mark specific parts of lesions; and assist drug injections to treat some diseases, such as dystonia. Injecting the botulinum toxin into convulsive muscles under the guidance of US has proven to be an effective treatment to reduce muscle tone (Dressler et al., 2021). However, in some patients, botulinum toxin injections in clinical studies have shown low efficacy. The main reasons for this observed lack of efficacy are inaccurate injection location or inappropriate dose of the botulinum toxin. The botulinum toxin must be precisely injected into the target muscle, which is essential to reduce adverse reactions in adjacent muscles and to achieve the maximum therapeutic effect at the lowest possible dose (Jinnah et al., 2016; Walter and Dressler, 2014). Identification of the muscle boundary plays a very important role in accurate injections. At present, the identification and marking of muscle boundaries by US is mainly achieved manually, which requires a lot of manpower, time, and experience. Muscle boundary recognition through US scans requires the operator to have rich professional knowledge and clinical experience. Therefore, such scans are mainly carried out by professional US doctors at present, which hinders the clinical application and popularization of US technology. In addition, there is no uniform standard for operators to mark muscle boundaries, and the subjective judgment of the operator can have an impact on the US images obtained. The use of different US equipment may also impact the images obtained from the manual marking of muscle boundaries (Cronin et al., 2020; Wijntjes and van Alfen, 2021).

**FIGURE 1:**
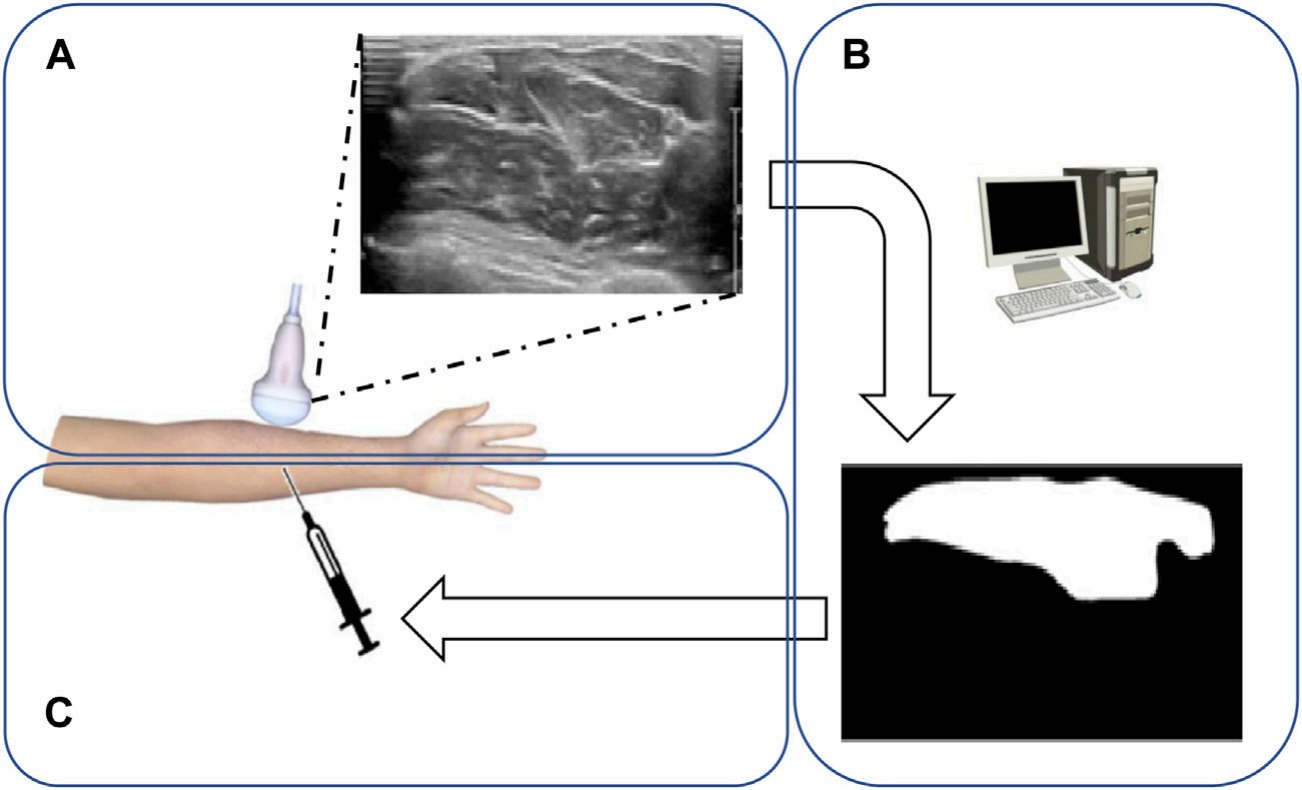
Flowchart of forearm muscle boundary detection from ultrasound datasets. **(A)** Doctors use the ultrasonic detector to scan the upper arm to get the muscle ultrasonic image. **(B)** Computers calculate and segment the muscle region through suggested models. **(C)** Doctors perform muscle injections according to the obtained muscle region.

In recent years, artificial intelligence has evolved and deep learning has become the leading machine learning tool in various research fields, especially in general imaging analysis (including natural and medical image analyses) and computer vision (Chan et al., 2020). The use of deep learning in US image analysis is also a growing trend (Liu et al., 2019; Marzola et al., 2021; Shen et al., 2021). Medical image segmentation is a technology that can label the boundary and shape of human tissues and organs. Traditional image segmentation methods are usually based on region segmentation and boundary segmentation. In 2015, full convolutional networks (FCNs) performed semantic segmentation in an end-to-end form (Shelhamer et al., 2017). FCNs can input images of arbitrary sizes, avoiding the problems of repeated storage and computational convolution caused by the use of pixel blocks. However, it is cumbersome to train and does not make full use of global contextual information, and its segmentation accuracy is insufficient. U-net is an improved network for FCNs. U-net is flexible and simple, and can obtain good segmentation from few sample datasets. It can also make better use of the global contextual information and the effective integration of low- and high-level information (Ronneberger et al., 2015). In addition, there are many other excellent models that can be used for image segmentation, such as DeepLabv3+, PSPNet, and Mask R-CNN (Chen et al., 2018; He et al., 2017; Zhao et al., 2016). DeepLabv3+ and CNN have a strong boundary detection capability, which can extract more detailed features and obtain better segmentation results. However, they have more parameters and are more computationally intensive, thus requiring higher-end equipment, larger sample data size, and longer training time. At present, U-net has become a mainstream method of medical image segmentation. Based on the U-net model, we have developed a tool for automatic muscle segmentation. This tool automatically identifies and objectively analyzes muscle boundaries in US images to inform and monitor the diagnosis and treatment of musculoskeletal disorders (Figure 1). The purpose of this study was to evaluate the feasibility of the application of this tool in clinical diagnosis, treatment, and teaching. We aim to ultimately help clinicians obtain muscle boundary images using dynamic US, shorten the learning period for using US, and promote the popularization and application of US-guided technologies.

## 2 Materials and methods

### 2.1 Participants

After receiving a detailed explanation of the purpose and potential risks of the experiment, all participants provided their written informed consent. The study protocols have been approved by the Medical Ethics Committee of Qilu Hospital, Shandong University (approval number: KLY-2020 (KS)-477). The study was carried out in accordance with relevant guidelines and regulations. The inclusion criteria were as follows: 1) participants were aged 20–50 years; 2) participants were healthy and did not have any cardiovascular or musculoskeletal diseases. The exclusion criteria were as follows: 1) any history of forearm surgery, forearm injury, or any pain in the forearm; 2) history of peripheral nerve injury; 3) history of thyroid or autoimmune diseases; 4) inability to cooperate with the study protocol. Nineteen participants enrolled in this study, who were aged 20–40 (27.4 ± 6.3) years. The participants included 10 men and 9 women, whose average body mass index (BMI) was 24.9 and 24.1 kg/m^2^, respectively.

### 2.2 Experimental procedure

Dynamic US images of the flexor digitorum superficialis (FDS) were acquired by a physiatrist with 3 years of experience in musculoskeletal US, using a 4–18 MHz linear transducer (EPIQ 7, Philips, Netherlands). All participants were positioned with the palm facing upward and their wrist in a neutral position. The transducer was first placed at the level of the proximal tendon of the FDS. During the process of the transducer sliding to the wrist, the FDS muscle was always kept identified in the transverse view and could be clearly observed by US dynamic imaging. Every video was about 10 s, and video clips within 200 frames-per-second were recorded.

Sequential images were picked at intervals of three or five frames from the videos. The resolution of each ultrasonic image was 707 × 346, and the size of each ultrasonic image was 50 kb–70 kb. The muscular boundaries of the FDS were manually labeled by another physiatrist with musculoskeletal US expertise and a junior physician of a rehabilitation department using ITK-SNAP software (Figures 3A, B).

### 2.3 U-net model

In our study, we used the U-net model proposed by Ronneberger et al. (2015).

The model contains two parts, an encoder and a decoder (Badrinarayanan et al., 2017; Noh et al., 2016). The encoder path downsamples the input image by successive pooling and convolution operations to extract semantic information, while the decoder path progressively upsamples and combines high-level features with low-level features provided by the encoder path. The encoder consists of the repeated application of two 3 × 3 convolutions, each followed by a rectified linear unit (ReLU) and a 2 × 2 max pooling operation with stride 2 for downsampling. The decoder consists of upsampling of the feature map, followed by a 2 × 2 convolution (“up-convolution”) that halves the number of feature channels, a concatenation with the correspondingly cropped feature map from the contracting path, and two 3 × 3 convolutions, each followed by a ReLU. The model is named “U-net” because the decoder path is symmetric to the encoder path, which results in a U-shaped architecture. Unlike FCNs, the skip connection approach is used in U-net, which combines high-level features with low-level features (Wu et al., 2021).

**FIGURE 2:**
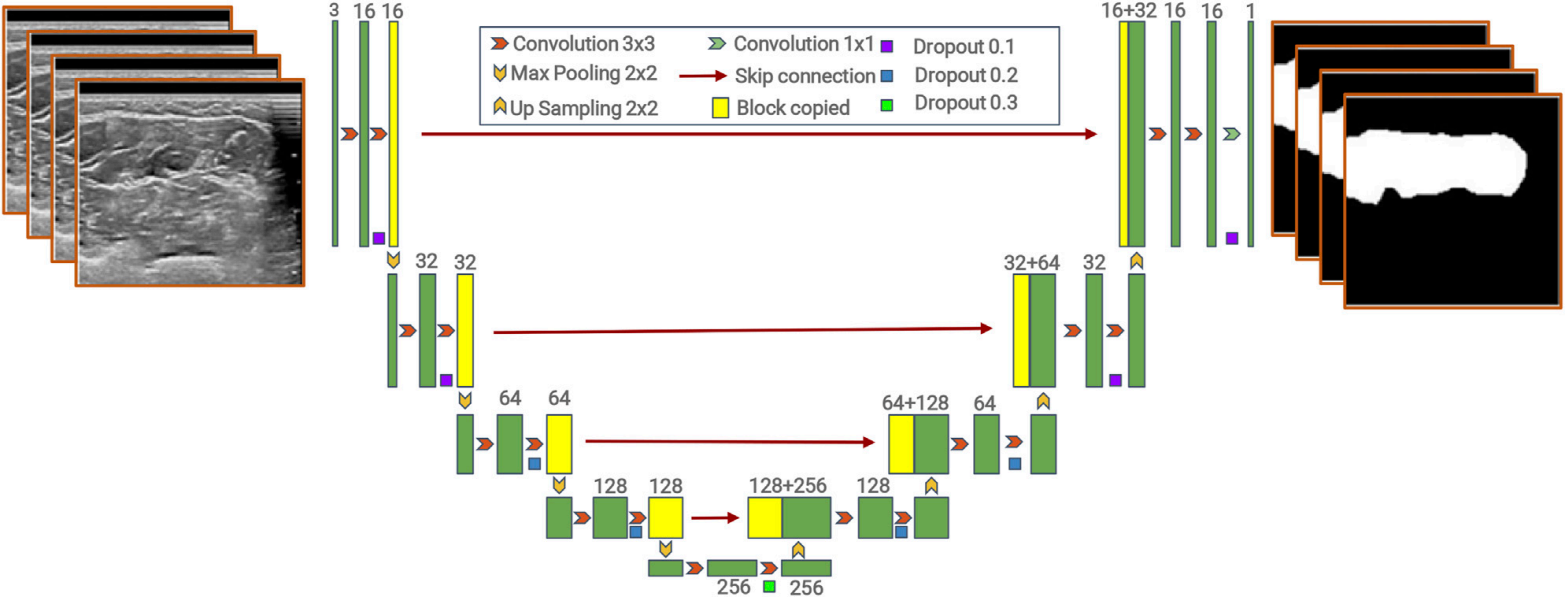
Architecture of the U-net structure.

The details of the model are shown in Figure 2.

### 2.4 Loss function

The loss function is BCELoss (binary cross-entropy loss). It can be used to solve the question of the multi-label classification, which is represented as follows:

$$Loss=-\frac{1}{N}\times \sum \left(y_{n}\right.\times \ln\left(x_{n}\right)+\left(1-y_{n}\right)\times \ln\left({1-x}_{n}\right),$$ (1)

where N represents the total number of pixels in an image, $y_{n}$ represents the true value, and $x_{n}$ represents the predicted value.

### 2.5 Evaluation metrics

Many criteria are used to measure segmentation results (Cordts et al., 2016). We selected four common standards in the field of image segmentation to analyze the results of our experiment.

The Dice coefficient is expressed as follows:

$$Dice\left(X,Y\right)=\frac{2\left|X\cap Y\right|}{\left|X\right|+\left|Y\right|}.$$ (2)

X is the area that is predicted to be the muscle, and Y is the targeted muscle area.

The higher the Dice score is, the better the segmentation performance is.

Precision is represented as follows:

$$Precision\;\left(X,Y\right)=\frac{\left|X\cap Y\right|}{\left|X\right|}.$$ (3)

X is the area that is predicted to be the muscle, and Y is the targeted muscle area. A higher value of Precision means that the predicted muscle area has a greater probability of being the true targeted muscle area.

Recall is represented as follows:

$$Recall\;\left(X,Y\right)=\frac{\left|X\cap Y\right|}{\left|Y\right|}.$$ (4)

X is the area that is predicted to be the muscle, and Y is the targeted muscle area.

Recall represents how much of the targeted muscle area is found in the prediction.

IoU is expressed as follows:

$$IoU\;\left(X,Y\right)=\frac{\left|X\cap Y\right|}{\left|X\cup Y\right|}.$$ (5)

X is the area that is predicted to be the muscle, and Y is the true muscle area.

The intersection over union (IoU) integrated the precision and recall, so it is used as a general indicator in many image segmentation tests. The higher the IoU score is, the better the segmentation performance is.

### 2.6 Post-processing

**FIGURE 3:**
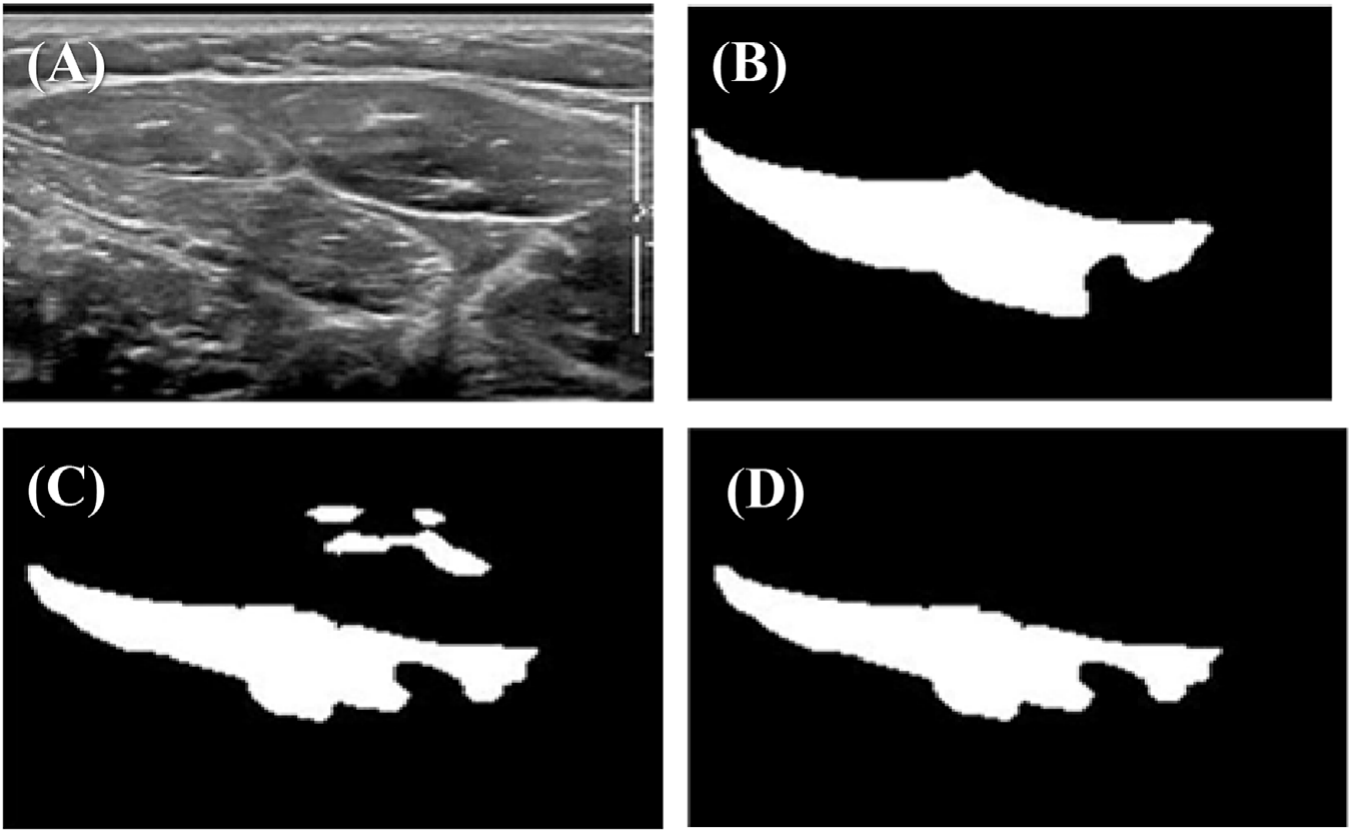
Example of outputs before and after post-processing: **(A)** original ultrasonic image; **(B)** true label marked by an expert sonographer; **(C)** prediction by U-net; **(D)** output after post-processing.

During the experiment, we found that the U-net output often included several disconnected regions (Figure 3C). However, anatomically, the target muscle usually only has one connected region. Based on this principle, we designed an algorithm to eliminate redundantly connected regions of outputs (Figure 3D). This algorithm is written in C++, and it is an application of the breadth-first traversal method of graph analysis. More specifically, this algorithm traverses all the points in the picture and counts out a total of several connected components, only retaining the largest connected component.

## 3 Results

### 3.1 Division of the dataset

The total number of US images obtained was 465, and the total number of marked images was also 465. In order to ensure stability, we conducted five experiments in parallel. In each experiment, all 465 images were divided into three parts, 405 of which were used for training, 30 of which were used for validation, and 30 of which were used for testing.

### 3.2 Experimental parameters

In our experiments, we used the TITAN X GPU (24 Gb) to accelerate the training procedure. The experiments were implemented using Python with PyTorch. We used the Adam optimizer (params, lr = 0.001, betas = (0.9, 0.999), eps = 1e-08, and weight_decay = 0). The epoch was 160, and the batch size was 32, and we chose the best model from the validation set as the final model.

### 3.3 Results

Table 1 and Table 2 detail the statistical characteristics of the prediction set in five groups.

**TABLE 1:** Average precision, recall, Dice coefficient, and IoU of the predicted set by U-net in five groups. Each item in the table is in the following form: average value (min value ∼ max value).

	Precision	Recall	Dice	IoU
Group 1	89.8% (57.5%–98.9%)	91.7% (58.3%–99.6%)	90.2% (69.9%–97.9%)	82.9% (53.7%–95.9%)
Group 2	92.0% (73.1%–98.4%)	92.0% (72.4%–98.6%)	91.8% (78.6%–96.3%)	85.1% (64.8%–93.0%)
Group 3	91.4% (73.1%–98.4%)	92.6% (77.7%–98.4%)	91.9% (84.7%–95.8%)	85.2% (73.5%–91.9%)
Group 4	91.7% (77.2%–98.3%)	92.7% (77.9%–98.3%)	92.0% (84.8%–96.5%)	85.0% (73.7%–93.3%)
Group 5	92.6% (76.9%–96.9%)	91.4% (53.0%–98.4%)	91.7% (66.7%–97.7%)	85.2% (50.1%–95.5%)
Average	91.50%	92.08%	91.52%	84.68%

**TABLE 2:** Average precision, recall, Dice coefficient, and IoU of the predicted set by the junior physician in five groups. Each item in the table is in the following form: average value (min value ∼ max value).

	Precision	Recall	Dice	IoU
Group 1	76.1% (1.1%–98.1%)	70.8% (1.6%–98.6%)	71.5% (1.2%–96.1%)	60.1% (0.6%–92.1%)
Group 2	68.7% (1.0%–99.2%)	71.1% (0.4%–97.1%)	68.0% (1.0%–93.2%)	57.5% (0.5%–88.4%)
Group 3	66.4% (0.2%–99.1%)	75.2% (0.4%–97.3%)	71.1% (0.3%–95.1%)	60.6% (1.0%–91.7%)
Group 4	75.3% (2.5%–98.1%)	77.0% (3.0%–97.5%)	74.2% (2.8%–95.3%)	64.1% (1.4%–91.8%)
Group 5	74.5% (0.5%–98.6%)	74.1% (0.1%–98.1%)	72.0% (0.06%–96.4%)	62% (0.3%–93.4%)
Average	71.80%	73.56%	71.20%	60.72%

The results given in the tables show that the five groups of experiments have little differences and high values, reflecting robust experimental results.

In order to assess the accuracy of the results more intuitively, we asked a junior physician of the rehabilitation department to perform manual segmentation on the same prediction set. The segmentation results are shown in Table 2.

Comparing the results obtained by the U-net model by manual segmentation by the junior physician, we found that the average intersection over union of the prediction set by U-net was 84.68%, while the average IoU of the prediction set by the junior physician was only 60.72%. In addition, the average precision, recall, and Dice coefficient of the prediction set by U-net were about 90% each; however, the average precision, recall, and Dice coefficient of the prediction set by the junior physician were only about 70% each. This demonstrates that the segmentation performance of the junior physician was inferior to that of U-net, and the ability of computers to identify targeted muscle areas was significantly higher than that of junior doctors.

### 3.4 Clinical application

As a concise explanation, the prediction of a model is considered good if the IoU value is high. On the contrary, the prediction is considered poor if the IoU value is low.

Initially, we compared the best two predictions given by the U-net model with the best two predictions by the junior physician.

**FIGURE 4:**
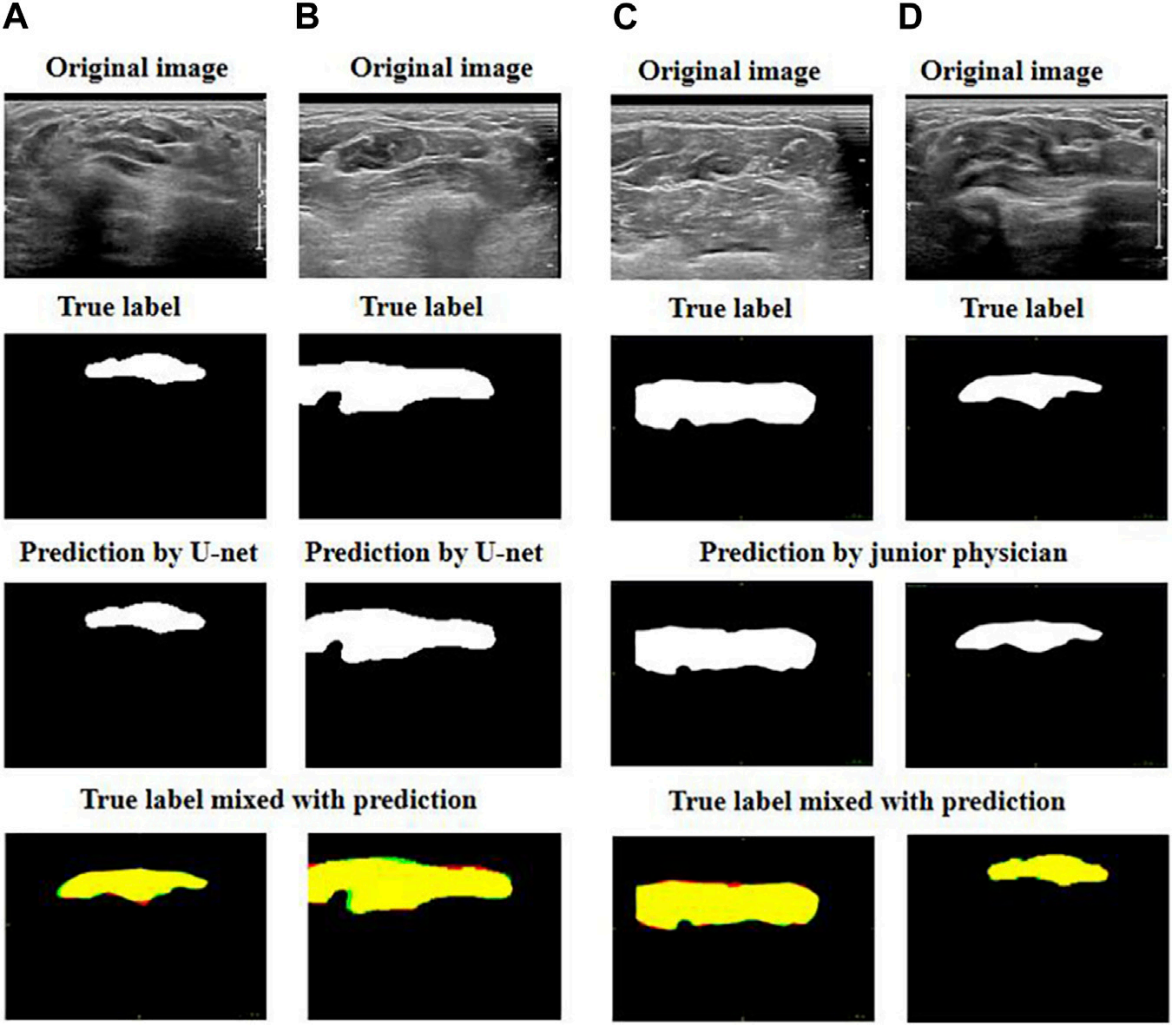
Exhibition of good prediction cases: **(A)** and **(B)** are the best predictions by U-net; **(C)** and **(D)** are the best predictions by the junior physician. The four images can be explained as follows: red represents the target muscle, green represents the predicted muscle, and yellow represents the intersection of red and green (i.e., yellow represents the intersection of the target muscle and the predicted muscle).

Figure 4 demonstrates that the best two predictions by U-net and by the junior physician were both good as their predictions were very close to the targeted muscle area.

**FIGURE 5:**
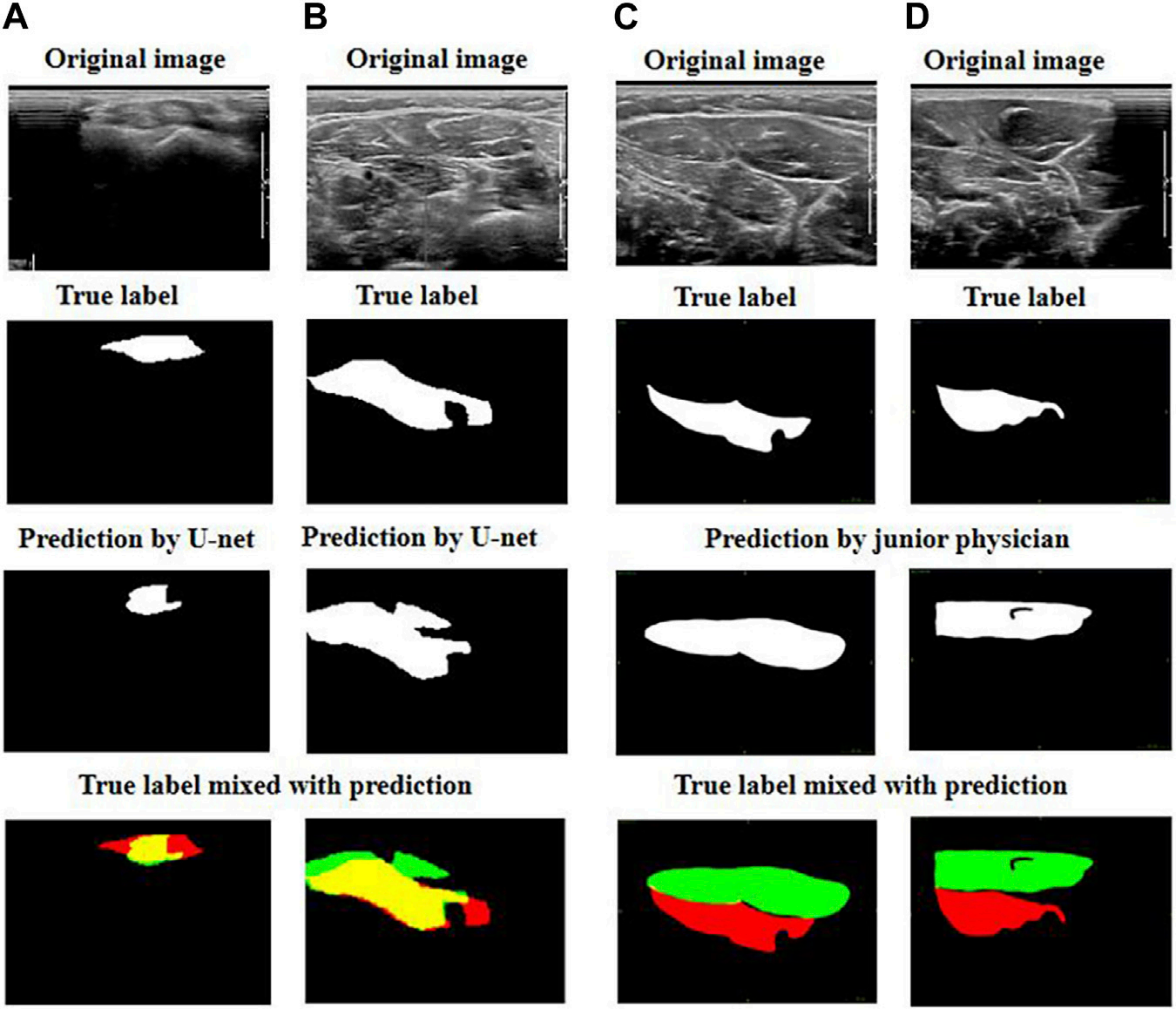
Exhibition of poor prediction cases: **(A)** and **(B)** are the worst predictions by U-net; **(C)** and **(D)** are the worst predictions by the junior physician. The four images can be explained as follows: red represents the target muscle, green represents the predicted muscle, and yellow represents the intersection of red and green (i.e., yellow represents the intersection of the target muscle and the predicted muscle).

We then focused on comparing the two worst predictions by the junior physician and the U-net model (Figure 5).

We observed that when the junior physician encountered US images with multiple muscle regions, they had more difficulty recognizing the boundary between the target muscle and other muscles or non-muscle tissues. If a muscle injection is performed in these conditions, the consequences are likely to be out of control.

However, although the predictions of our U-net model do not fit the edge contours perfectly, the predicted area must have a significant overlap with the targeted muscle. When faced with multiple muscle-like regions in US images, our U-net model can accurately identify the target muscle.

During the muscle injection process, by combining the U-net prediction with the real US image, doctors can accurately recognize the target muscle. Therefore, the model is perfectly useful for doctors in clinics.

In order to verify our results more rigorously, we assessed the accuracy of the “middle point” prediction. Here, we define an excellent prediction as one where the “middle point” of the prediction belongs to the target muscle.

The “middle point” can be defined as follows:

$$the\;x\;coordinate\;of\;“middle\;point”=\frac{\left(Xmin+Xmax\right)}{2},$$

$$the\;y\;coordinate\;of\;“middle\;point”=\frac{\left(Ymin+Ymax\right)}{2}.$$

Xmin and Xmax are the x coordinates of the leftmost and rightmost points predicted to belong to the target muscle.

Ymin and Ymax are the y coordinates of the lowest and uppermost points predicted to belong to the target muscle.

For the 150 images predicted by the junior physician, 127 can be regarded as excellent. However, out of the predictions by the U-net model, 149 can be regarded as excellent. In other words, 18% of the images confused the junior physician, while the probability of misjudgment by the U-net model is less than 1%.

## 4 Discussion

The current intramuscular injection of drugs for the treatment of musculoskeletal diseases is mainly achieved by direct injections, electromyography-guided injections, or US-guided injections. Direct injections greatly depend on the clinician’s knowledge of anatomy and clinical experience; the risks of the injection are high and its accuracy is not guaranteed. Electromyography-guided injections are invasive and could cause pain and discomfort in patients. US has the advantages of being non-invasive, portable, low cost, easy to operate, and has no associated radiation. Most importantly, US-guided injections have the capability for real-time imaging, allowing for the monitoring of continuous dynamic images. However, US images have the disadvantages of low clarity and resolution, and the target tissue may not contrast well with the surrounding tissue (Pillen et al., 2016). Therefore, artificial recognition in US images is difficult and the learning cycle for US practitioners is lengthy. Hence, an auxiliary tool is needed to shorten the US learning cycle for clinicians.

Deep learning has been widely used in various fields of medicine. Current research related to deep learning has involved the recognition and segmentation of various anatomical structures, such as the liver, breast, and thyroid in medical imaging, and the diagnosis and recognition of pathological changes in tumors (Jiang et al., 2020; Poudel et al., 2018; Guo et al., 2021; Mishra et al., 2019). Our research applies the U-net model for medical image segmentation to assist in the treatment of various musculoskeletal disorders, and achieve good training results and segmentation recognition accuracy with few sample datasets.

In this study, we compared the muscle boundary US images identified by our newly developed automatic muscle segmentation tool with the manually labeled muscle boundary US images of junior and senior practitioners, respectively. It was found that the accuracy of the muscle boundary US images obtained by the automatic muscle segmentation tool was very similar to those manually labeled by a specialist sonographer and was significantly higher than those labeled by a junior practitioner. Thus, we believe that the accuracy of this automatic muscle segmentation tool can be trusted. The use of this automatic muscle segmentation tool by clinicians can save a great deal of time, reduce the clinician’s workload, and improve injection safety. It also reduces the dependence on the professional knowledge and experience of the operator, who only needs to be familiar with the operation of this tool to automatically identify and mark muscle boundaries. This will facilitate the spread and application of US-guided technologies in clinical practice. This tool could also enable continuous identification and labeling in moving images, reducing the difficulty of manually labeling moving images and facilitating clinical analysis. Furthermore, this tool will help in avoiding damage to blood vessels and nerves during injections. This automatic identification also provides the possibility of identifying muscles and other tissues, and therefore, it may be used as a novel diagnostic tool for peripheral nerve diseases, which is easier to obtain and operate with (Ali et al., 2016; Ozçakar et al., 2012).

However, the current work contains several limitations. First, in comparing the images labeled by professional sonographers with those automatically recognized by the tool, we found that there is still a lack of fine recognition of muscle boundaries. This may be due to our small sample size. Future studies must focus on increasing the number of sample images to achieve better training results and, therefore, higher accuracy. Second, the sample images we obtained were from healthy participants and did not include patients with limb spasms. We should actively apply the tool to patients with musculoskeletal disorders in the future as well. Third, our current work has completed the identification of the boundaries of a single muscle in the forearm, and the identification of the boundaries of multiple muscles is needed to assist in the combined injection therapy of multiple muscle groups. Finally, the images were acquired by one physician using a particular US machine. This means that the model may overfit the features provided by the training datasets and may be less effective in the segmentation of images acquired by other types of US machines. Therefore, we need to add training datasets from different sources to improve the practicability of the model (Wu et al., 2021).

## 5 Conclusion

In our study, we have developed an automatic muscle segmentation tool for US-guided muscle injections. We have demonstrated that the accuracy of the recognition of the muscle boundary of the FDS in an US image by the automatic muscle segmentation tool was similar to those manually labeled by a specialist sonographer and was significantly higher than that by a junior physician. We provide a reliable auxiliary tool for clinicians to shorten the US learning cycle, reduce clinical workload, and improve injection safety. In the future, the average living standard of human beings will rise significantly and the age of the aging population will arrive. Even though the healthcare system has developed rapidly, it is still unable to meet the huge demand for medical resources. In order to improve the efficiency of medical decision-making, the medical image segmentation technology based on deep learning is bound to flourish. On the basis of model and algorithm updates and iterations, we should continue to explore the direction from simple diseases to complex diseases and from 2D segmentation to 3D segmentation.

## Data Availability

The raw data supporting the conclusion of this article will be made available by the authors, without undue reservation.
